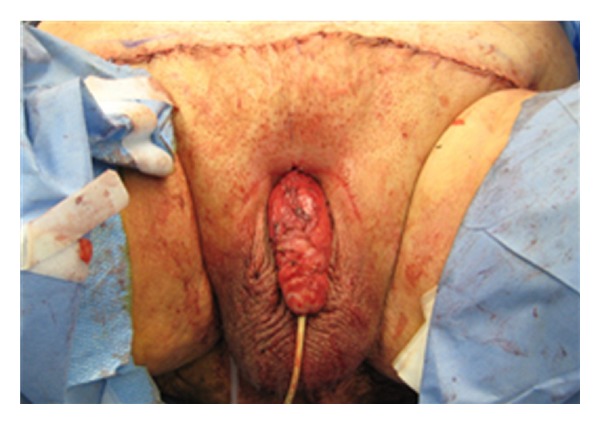# Penile Enhancement Procedures with Simultaneous Penile Prosthesis Placement

**DOI:** 10.1155/2012/314612

**Published:** 2012-06-28

**Authors:** Tariq S. Hakky, Jessica Suber, Gerard Henry, David Smith, Paul Bradley, Daniel Martinez, Rafael E. Carrion

**Affiliations:** ^1^Department of Urology, University of South Florida, 2 Tampa General Circle, Tampa, FL 33602, USA; ^2^Division of Plastic Surgery, University of South Florida, 2 Tampa General Circle, Tampa, FL 33602, USA; ^3^LSU Health Sciences Center, 255 Bert Kouns Industrial Loop, Shreveport, LA, USA

## Abstract

Here we present an overview of various techniques performed concomitantly during penile prosthesis surgery to enhance penile length and girth. We report on the technique of ventral phalloplasty and its outcomes along with augmentation corporoplasty, suprapubic lipectomy, suspensory ligament release, and girth enhancement procedures. For the serious implanter, outcomes can be improved by combining the use of techniques for each scar incision. These adjuvant procedures are a key addition in the armamentarium for the serious implant surgeon.

## 1. Introduction

Penile length and girth has long been a source of anxiety for men and still is today. Through the ages men have undergone a myriad of different measures for penile enhancement. Historically, holy men in India and Cholomec tribesmen in Peru used weights to increase penile length, while Dayak tribesmen in Brazil allowed poisonous snakes to bite their penises to enlarge them [1]. Men often feel a need to optimize their penile dimensions in order either to improve their self-esteem and or to impress their partners. In the modern era, a significant number of men who have radical surgery will suffer from loss of stretched penile length from 0.5 to 5 cm [1, 2]. Additionally, apparent loss of length occurs in many men as a consequence of weight gain in which the penis is “buried” under the excess skin of a panniculus.

Penile prosthesis surgery is a widely accepted treatment for men with erectile dysfunction refractory to pharmacologic therapy. It is associated with satisfaction rates of greater than 90% [3]. In patients requiring implantation of a penile prosthesis many report that their prosthetic erection is shorter than their former natural erection [1–4]. Different strategies have been implemented in order to increase phallic length. This includes penile rehabilitation such as oral phosphodiesterase-5 inhibitors, muse, vacuum erection devices, and intercavernosal injections. Several intra-operative techniques such as ventral phalloplasty, corporoplasty, and suprapubic lipectomy have been described as intraoperative techniques to improve phallic length.

Through simple adjuvant procedures, it is possible to maximize the perception of size concomitantly during penile prosthesis surgery. To enhance patient perception of penile length, it is feasible to perform simple procedures that will increase perceived or true penile length. Here we present an overview of various techniques, which improve the surgical armamentarium of the serious implanter. 

## 2. Ventral Phalloplasty

Up to 84% of patients who have undergone successful placement of penile prosthesis often complain of penile shortening [5]. To combat this one may take down the penoscrotal web to enhance patient satisfaction. The use of scrotoplasty has been described in pediatric literature to improve the projection of the webbed variant of inconspicuous penis [5]. 

**Figure 1:**
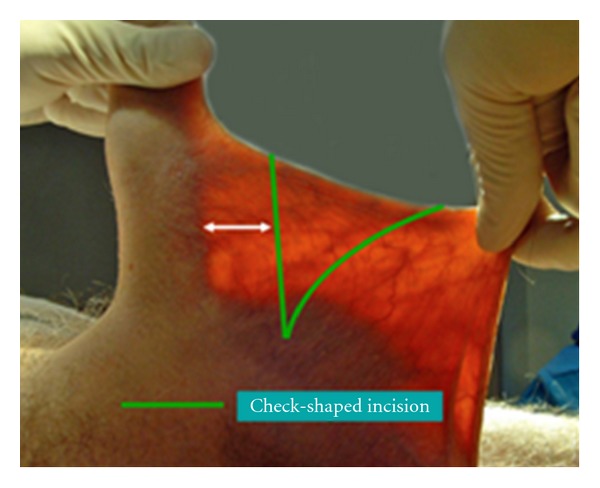


**Figure 2:**
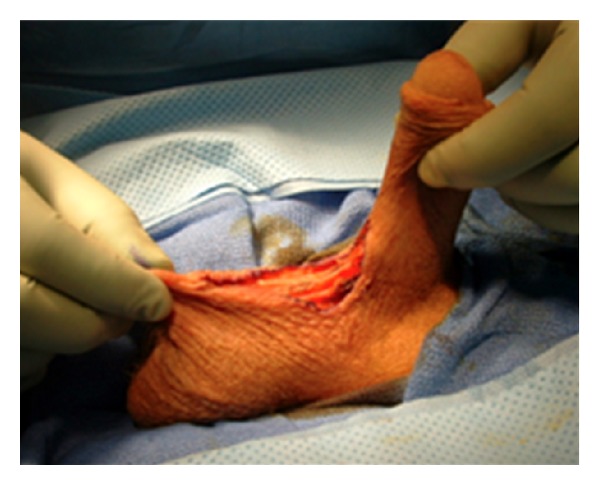


**Figure 3:**
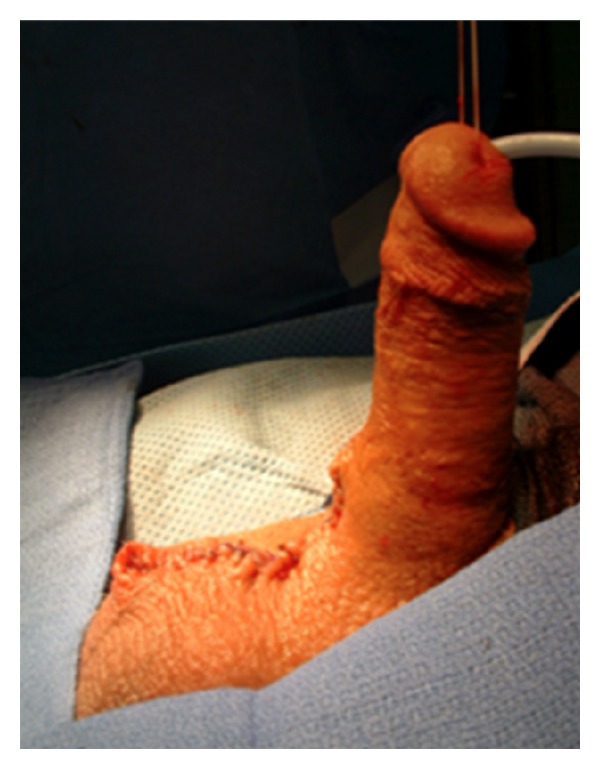


By holding the scrotum along the median raphe and stretching it out, one delineates the extent of the penoscrotal web. An Alice clamp may be used to assist in the elevation the penoscrotal web. One may enhance the dissection by placing a light source behind the web, causing a silhouette on the penile shaft and testicles clearly delineating the extent of the web (Figure 1). A “check mark” incision is marked [5]. Along the *Y* axis one marks at the incision line with one fingerbreadth's clearance from the shaft allowing for adequate skin closure. This line is carried down to the penoscrotal angle. At this point a convex curve is taken up to the scrotal skin resembling a “check mark” [4, 5]. This skin is removed leaving a diamond-shaped defect in the scrotum (Figure 2). A thick layer of dartos fascia is preserved to ensure adequate healing. The dartos is reapproximated with interrupted stitches along the axis of the shaft. The scrotal skin is brought together with interrupted horizontal mattress sutures (Figure 3).

As reported by our original investigation on a group of 43 patients undergoing phalloplasty and penile prosthesis placement, 84% of patients reported some increased degree of phallic length while 12% reported no significant change in penile length after phalloplasty [5]. Ventral phalloplasty can enhance patient perception of penile length and improve overall satisfaction and can concomitantly be performed during penile implant surgery [3–5]. Release of penoscrotal web is a simple, safe, and reproducible procedure that can enhance patient perception of penile length and further improve satisfaction. 

## 3. Augmentation Corporoplasty

The tunica albuginea is composed of elastic fibers, collagen, and has a wide network of perforating vessels. Corporoplasty is the ability to modulate the tunica albuginea. The most ideal graft should be elastic with minimal resistance and fibrosis. This patch grafting is commonly used in the setting of Peyronie's disease but can be an adjuvant in the setting of penile prosthesis placement. 

After placement of a penile prosthesis, several different corporal maneuvers can cause phallic shortening such as incorrect dilation or improper sizing of the penile prosthesis. Additionally, a high riding pump can also have a penile shortening effect. Corporal augmentation is the most direct method of elongating the phallic length. There are three main classes of biological material used for corporoplasty, which include human grafts, treated biological materials, and synthetic materials [6, 7]. Human venous grafting in the setting of corporoplasty avoids a fibrous reaction on the erectile tissue; however, it requires vascular support and therefore cannot come into direct contact with the prosthesis [6]. Additionally, there is patient morbidity associated with the harvesting of saphenous vein or dermis. Austoni et al. have described a method making incisions in the tunica albuginea and suturing a saphenous vein graft material into the created space [6]. Treated biological material such as AlloDerm or Tutoplast has decreased inflammatory activity that promotes natural tissue remodeling, with minimal fibrosis. There is little to no harvesting, and they are antigen-free with multidirectional collagen fibers yielding excellent tensile strength. Paradiso et al. implanted SIS in several penile prosthesis patients that demonstrated rapid attachment with minimal fibrosis and improved penile lengthening [8]. Various series have reported the use of synthetic products such as Gore-Tex or Silicone [6, 7]. However, the complication rate following penile grafts of these synthetic materials is high and includes a greater risk of fibrosis and the absence of elasticity. While synthetic graft always produces macrophage activity followed by intense fibroblasts activity, we favor pretreating our patients with vacuum erection device and intraoperative corporal molding at the time of penile prosthesis. In the setting of penile prosthesis placement, the implementation of these products can be a tricky. The use of biological material for corporoplasty requires more experience before it can be widely used in clinical practice. 

In patients with corporal fibrosis, Wilson et al. reported on the use of downsizing the penile prosthesis cylinders, which were then used as tissue expanders in patients [7]. During a 12-month period of intracorporal stretching, the patient was instructed to inflate the prosthesis for up to 3 hours a day. The resultant expansion of an average 2.2 cm was noted. The newly molded intracorporal cavity allowed for the subsequent placement of wider and longer implants [7]. While this sound enticing, it subjects the patient to yet another surgery, which can be simply avoided with the use of a preoperative vacuum erection device along side judicious intraoperative prosthesis oversizing.

## 4. Suprapubic Lipectomy

**Figure 4:**
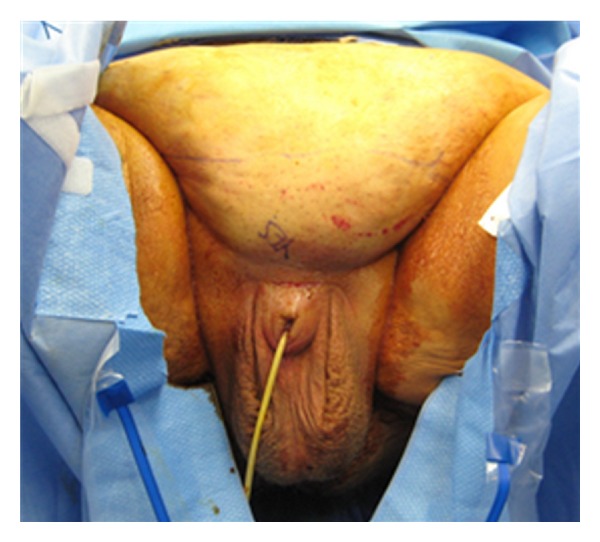


**Figure 5:**
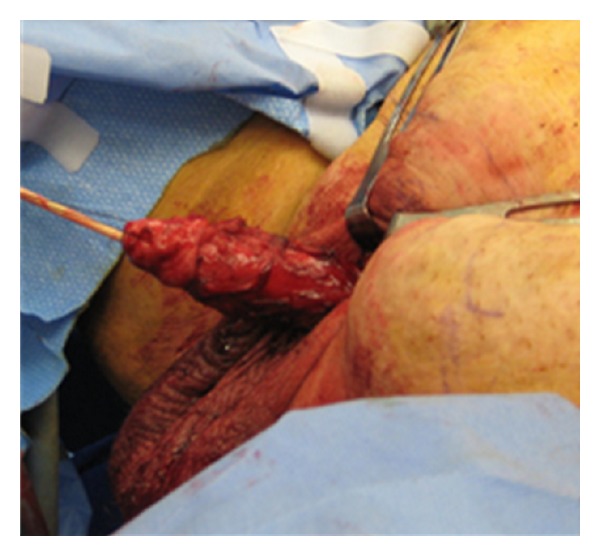


**Figure 6:**
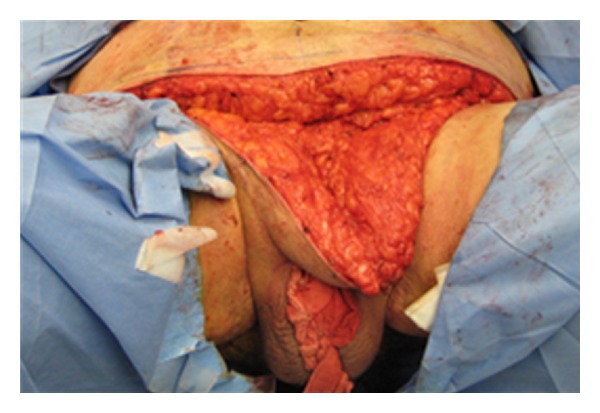


Apparent loss of length occurs in many men as a consequence of weight gain, in which the penis is buried under the excess skin of the panniculus. There have been a variety of techniques described for the treatment of buried penis. The surgical technique for buried penis was first described by Horton et al. [9]. Initially, the suprapubic fat is excised with release of the suspensory ligament of the penis and dartos fascia. At this point, the suprapubic skin is secured to the rectus fascia [9]. Recently, panniculectomy with suction-assisted lipectomy and anchoring of herniated pubic skin to the abdominal wall has been utilized with satisfactory results. The technique includes preoperative marking of the patient in the standing position as the landmarks of resection and amount of pannus to be removed become obscured in the supine position (Figure 4). The suprapubic area and lower abdomen are then infiltrated with tumescent solution. Suction lipectomy is performed (Figure 5) in this area with care to protect the testicles and spermatic cords. Next, the panniculectomy is performed (Figure 6). The suspensory ligament may be separated if indicated. After excision of excess skin and fat, the pubic skin and base of the penis are sutured to the rectus fascia. The wound is then closed in a layered fashion over a drain (Figure 7). Postoperatively, a pressure garment may be worn for 4–6 weeks [10]. Other techniques described in literature include Z plasties with circumcision, release of dartos tethering bands, pedicled preputial flaps, and/or split-thickness skin graft to the penile shaft with vacuum-assisted closure negative pressure dressing [11]. Addressing this issue in patients involves the coordination and planning between the plastic surgeon and urologic surgeon. Each patient will require a personalized plan and may require a modification of or combination of any of the above-mentioned procedures [11]. For the serious implanter, this can be challenging, but in a team setting along side the plastic surgeons this technique can increase patient satisfaction. Suprapubic lipectomy can give the morbidly obese patient an improved sex life safe and feasible in the team setting.

## 5. Suspensory Ligament Release

The penile suspensory ligament is composed of the suspensory ligament and the arcuate ligament. The ligation of the penile suspensory ligament permits the penis to drop into a more dependent position. This gives the patient a perceived gain in phallic length; on average this procedure adds 1 cm of flaccid penile length. The technique most commonly used for releasing the suspensory ligament is in combination with an inverted V-Y skin plasty; however, V-Y half-skin half-fat advancement flap and T closure have also been reported [11]. After ligation, it is essential that a weight or stretch device be used as failure to do so can lead to reattachment of the ligament and possible decrease in phallic length [12, 13]. Average length gained in certain series was 2.4 cm with motivated patients gaining up to 3.2 cm [12, 13]. Insertion of a silicone buffer has been reported, and the placement of this spacer is used to prevent reattachment [13]. Borges et al. performed suspensory ligament release in 303 patents at the time of penile prosthesis placement. They report a 93% satisfaction with penile prosthesis performance and penile length. Additionally, they demonstrated that none of their patients reported penile shortening [14].The release of the suspensory ligament is a quick simple procedure with minimal patient morbidity that is another tool for the serious implanter to gain penile length during concomitant penile prosthesis placement. Alongside the risk of reattachment this adjuvant procedure may mean a second incision if penile prosthesis placement is performed from a peno-scrotal approach. 

## 6. Girth

Various materials have been tried to increase phallic girth that include but are not limited to paraffin, mercury, silicon, petroleum jelly, stone, and cod liver oil [14]. These materials can cause foreign body reaction, scarring, deformity, and sexual dysfunction. We do not recommend their use with concomitant penile prosthesis surgery. Recently, Al-Ansari et al. reported on the application of a thigh flap with a vascular pedicle from the superficial circumflex iliac artery as a means to increase in penile girth through augmentation [15]. They noted an 8 cm increase in erect girth after augmentation [15]. While this surgery is a monumental reconstructive effort, it creates considerable increase in girth. However, the thigh flaps come at a high price to the patient with risks of graft loss and wound infections, and this would obviate the need for revision which is especially grave in the setting of a penile prosthesis. For the serious implanter, this technique may be of benefit; nonetheless, the risk benefit ratio is one to be questioned. We would not routinely recommend this technique unless the implanter is comfortable with safely mobilizing the vascular flap.

More recently, autologous tissue engineering and biodegradable scaffolds have been used as a new option to increase penile girth. After cells are harvested and cultured on a pretreated tube shaped, it is transplanted between dartos and Buck's fascia. Reports have shown an average gain in girth ranging from 1.9 to 4.1 cm [16]. More prospective controlled analyses are needed to help protocoled girth enhancement techniques. While many of these practices have yet to reach the mainstream, this is a future avenue for penile prosthesis placement in patients who lack girth. 

## 7. Conclusions

With an estimated 20,000 penile prosthesis placed every year, the potential for actual and perceived loss in penile length is apparent [17]. The implementation of simple adjuvant surgical procedures may increase phallic length. These techniques increase patient satisfaction when performed in concert with penile prosthesis placement and promote the perception of increase phallic length. Over the years, multiple surgical approaches have been suggested to facilitate this difficult situation. Approaches include ventral phalloplasty, augmentation corporoplasty, suprapubic lipectomy, and suspensory ligament release. At this time we do not recommend routine implementation of girth enhancement during penile prosthesis placement due to lack of protocoled enhancement techniques. For the serious implanter, outcomes can be improved by combining the use of techniques for each scar incision, for example, performing a suspensory ligament release during an infrapubic penile prosthesis placement. Surgical strategies like upsizing prosthesis and intraoperative molding, alongside suspensory ligament release, phalloplasty, or suprapubic lipectomy must be kept in mind as adjuvant procedures in the different approaches to the placement of penile prosthesis.

## Figures and Tables

**Figure 7:**